# Crystal structure of *N*,*N*′,*N*′′-tri­cyclo­prop­ylbenzene-1,3,5-tricarboxamide

**DOI:** 10.1107/S2056989024009800

**Published:** 2024-10-24

**Authors:** Manuel Stapf, Venugopal Rao Miyyapuram, Wilhelm Seichter, Monika Mazik

**Affiliations:** a Institut für Organische Chemie, Technische Universität Bergakademie Freiberg, Leipziger Str. 29, 09596 Freiberg/Sachsen, Germany; b Clinical Research Products Management Center (CRPMC) Bioservices Thermo Fisher Scientific, 1055 First Street Rockville/Maryland 20850 USA; Universidade de Sâo Paulo, Brazil

**Keywords:** crystal structure, hydrogen bonding, N—H⋯O bonds, C—H⋯O inter­action, benzene-1,3,5-tricarboxamide, cyclo­alkyl unit

## Abstract

The title compound, C_18_H_21_N_3_O_3_, was prepared from 1,3,5-benzene­tricarbonyl trichloride and cyclo­propyl­amine. Its crystal structure was solved in the monoclinic space group *P*2_1_/*c*. In the crystal, the three amide groups of the mol­ecule are inclined at angles of 26.5 (1), 36.9 (1) and 37.8 (1)° with respect to the plane of the benzene ring. The mol­ecules are linked by N—H⋯O hydrogen bonds, forming two-dimensional supra­molecular aggregates that extend parallel to the crystallographic *ab* plane and are further connected by C—H⋯O contacts. As a result of the supra­molecular inter­actions, a propeller-like conformation of the title mol­ecule can be observed.

## Abstract 2

In the crystal structure, the mol­ecules are connected by N—H⋯O hydrogen bonds to create two-dimensional supra­molecular networks extending parallel to the crystallographic *ab* plane.

## 1. Chemical context

The cyclo­propane ring represents a building block of numerous natural products and has also been recognized as a valuable structural motif in drug design (Reissig & Zimmer, 2003 ▸; Chen *et al.*, 2012 ▸; Talele, 2016 ▸; Wu *et al.*, 2018 ▸; Bauer *et al.*, 2021 ▸). Moreover, the cyclo­propyl group has also been used in supra­molecular chemistry, for example in the construction of artificial receptors (Stapf *et al.*, 2020 ▸). In this paper, we describe the crystal structure of a compound bearing *N*-cyclo­propyl­carbamoyl groups, which belongs to the class of benzene-1,3,5-tricarboxamides. Other representatives of this class of compounds, *e.g.* those with *N*-(pyridin-2-yl)carbamoyl or *N*-(1,8-naphthyridin-2-yl)carbamoyl groups, were found to have inter­esting binding properties towards carbohydrates (Mazik *et al.*, 2000 ▸, 2004 ▸, 2006 ▸; Mazik & Sicking, 2001 ▸, 2004 ▸; Mazik & Cavga, 2007 ▸). It is worth noting that various supra­molecular architectures based on benzene-1,3,5-tricarboxamide have been the subject of intensive research (Cantekin *et al.*, 2012 ▸). The self-aggregation processes of benzene-1,3,5-tricarboxamides have been studied particularly intensively and have led to the development of new hydro­gels, hydrogen-bonded organic frameworks (HOFs) and other systems with favourable properties (Stals *et al.*, 2009 ▸; Veld *et al.*, 2011 ▸; Howe *et al.*, 2013 ▸; Kulkarni *et al.*, 2017 ▸; Li *et al.*, 2024 ▸).
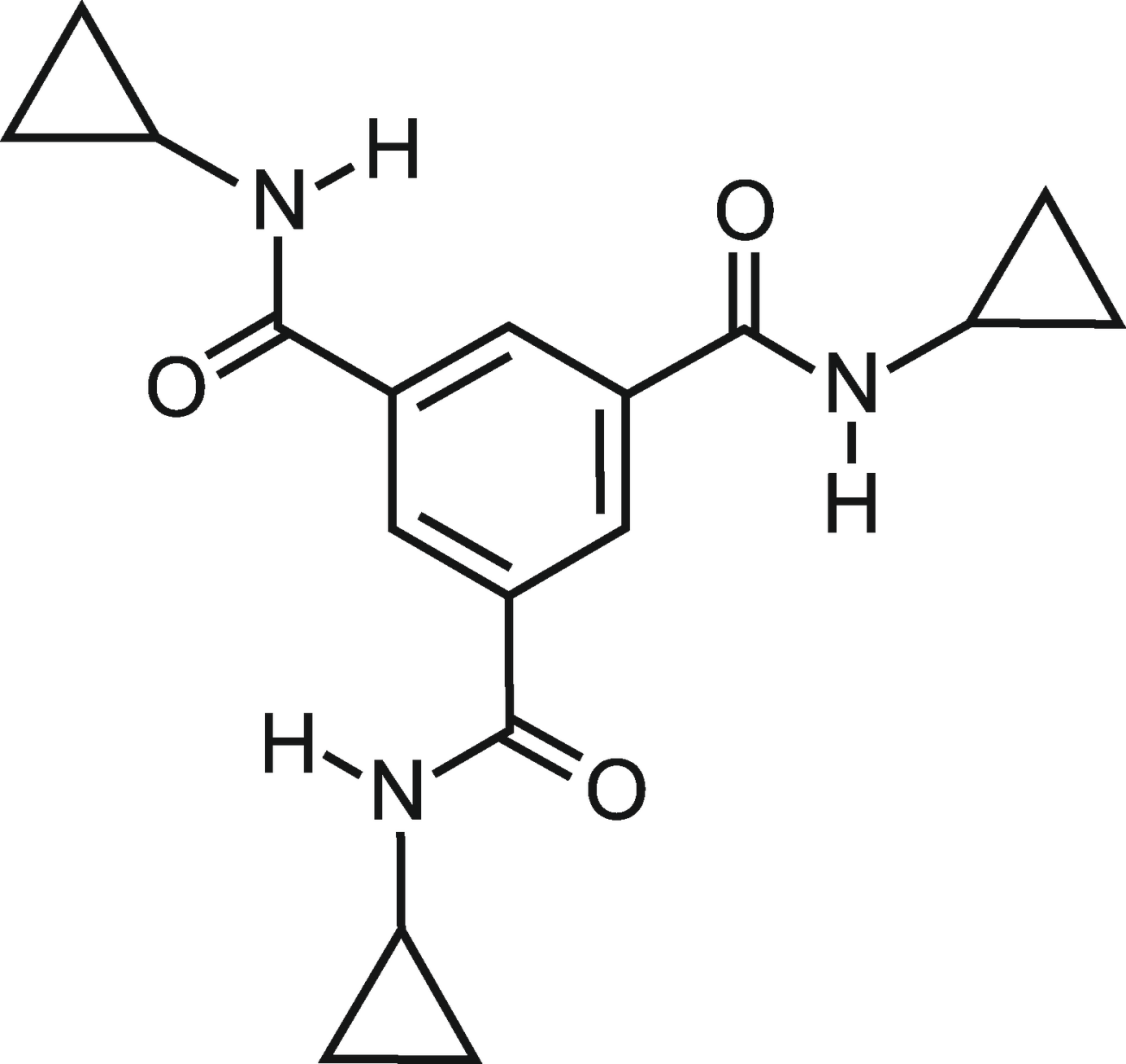


## 2. Structural commentary

**Figure 1:**
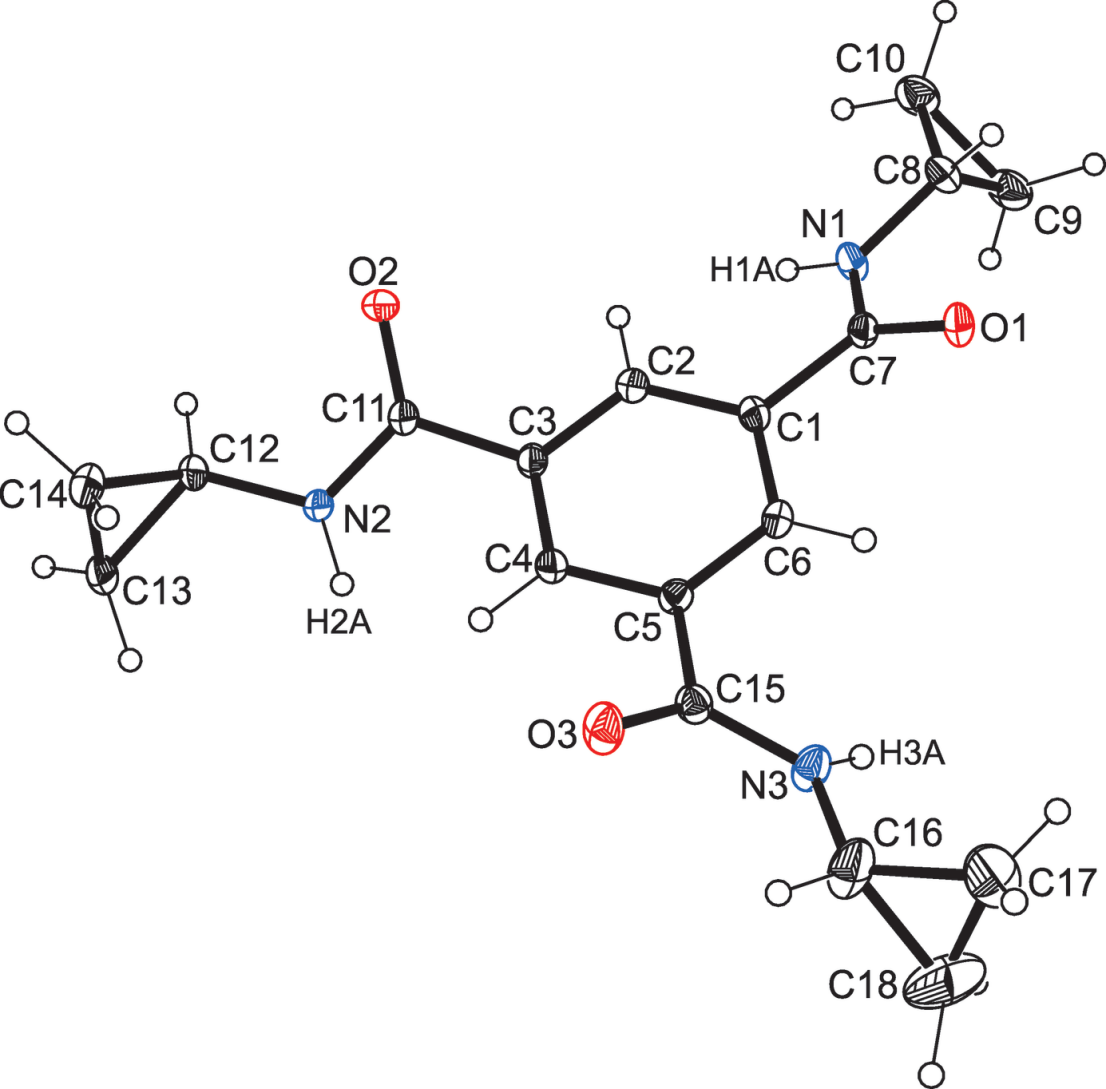
Perspective view of the title mol­ecule including atom labelling. Anisotropic displacement ellipsoids are drawn at the 50% probability level. For the sake of clarity, only the major component of the disordered cyclo­propyl ring is shown.

The crystal structure of the title compound, C_18_H_21_N_3_O_3_, was solved in the monoclinic space group *P*2_1_/*c* with the asymmetric unit containing one mol­ecule. One of the cyclo­propyl groups is disordered over two positions (s. o. f. 0.70/0.30). The three amide units of the mol­ecule are inclined at angles of 26.5 (1), 36.9 (1) and 37.8 (1)° with respect to the plane of the central benzene ring. This twisting, which is due to supra­molecular inter­actions, gives the threefold-substituted benzene derivative a propeller-like conformation (Fig. 1 ▸).

## 3. Supra­molecular features

**Figure 2:**
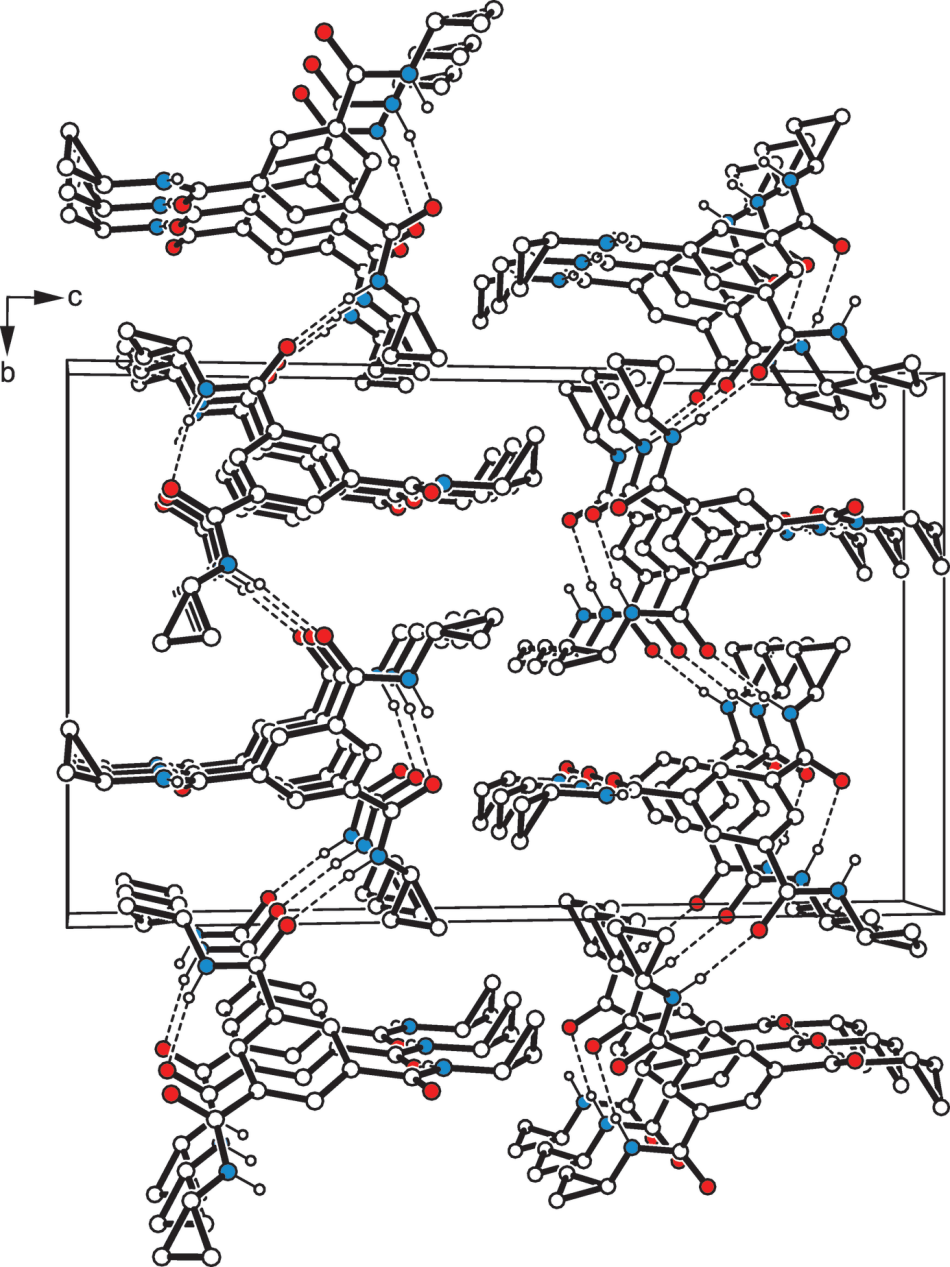
Packing diagram of the title compound viewed along the *a* axis. The N—H⋯O hydrogen bonds are visualized as dashed lines.

In the crystal structure of the title compound, the mol­ecules are connected by N—H⋯O bonds [*d*(H⋯O) 1.96 (1)–2.04 (1) Å, 159 (1)–168 (1)°; Table 1 ▸] to form two-dimensional supra­molecular networks extending parallel to the crystallographic *ab* plane (Figs. 2 ▸ and 3 ▸). Within these aggregates, the oxygen atom O3 participates in the formation of a C—H⋯O bond [*d*(H⋯O) 2.58 Å, 139°; for other examples of C—H⋯O bonds, see: Desiraju & Steiner, 1999 ▸; Desiraju, 2005 ▸; Mazik *et al.*, 1999 ▸, 2005 ▸, 2010 ▸; Ebersbach *et al.*, 2023 ▸] to the arene hydrogen H2 of an adjacent mol­ecule. Association of the 2D networks is accomplished by C—H⋯O bonds involving methyl­ene hydrogen H14*A* and the oxygen O1 [*d*(H⋯O) 2.55 Å, 131°].

## 4. Database survey

A search in the Cambridge Structural Database (CSD, Version 5.45, update June 2024; Groom *et al.*, 2016 ▸) for benzene derivatives containing at least one *N*-cyclo­alkyl­carbamoyl group with a cyclo­propyl, cyclo­butyl, cyclo­pentyl or cyclo­hexyl ring gave nineteen hits. Among these are fifteen *N*-cyclo­alkyl­benzamides, which also have other substituents on the benzene ring, such as hy­droxy, meth­oxy or halogeno groups, making comparison difficult. For example, compounds bearing both hy­droxy and meth­oxy groups and differing in ring size comprise *N*-cyclo­propyl-3-hy­droxy-4-meth­oxy­benzamide (HOBGOY; Tong *et al.*, 2023 ▸), *N*-cyclo­pentyl-3-hy­droxy-4-meth­oxy­benzamide (DELLUF; Zhang *et al.*, 2022*a* ▸) and *N*-cyclo­hexyl-3-hy­droxy-4-meth­oxy­benzamide (DELCUW; Zhang *et al.*, 2022*b* ▸). The crystal structure of the compound lacking further substituents on the benzene ring is only known in the case of the cyclo­hexyl unit (QUZJAX; Khan *et al.*, 2010 ▸). The same applies to compounds that have two or three carboxamide units on the benzene ring; these include *N*,*N*′-di(cyclo­hex­yl)benzene-1,4-dicarboxamide (DAVQUP01; Wang *et al.*, 2017 ▸) and *N*,*N*′,*N*′′-tris­(cyclo­hex­yl)benzene-1,3,5-tricarboxamide (CIYYAO; Li *et al.*, 2024 ▸). The latter, tripodal mol­ecule is an analogue of the title compound, but has a markedly different crystal structure. It consists of columnar domains extending in the direction of the crystallographic *b* axis, in which the mol­ecules are arranged in layers with a stacking of the central benzene rings. Neighbouring mol­ecules are mainly linked by three N—H⋯O=C hydrogen bonds. The periphery of the domains is formed by the cyclo­hexyl moieties, so that they are only connected to each other *via* van der Waals inter­actions.

## 5. Synthesis and crystallization

A solution of 1,3,5-benzene­tricarbonyl trichloride (0.20 g, 0.75 mmol) in CH_2_Cl_2_ (10 mL) was added dropwise to a mixture of cyclo­propyl­amine (0.18 mL, 0.15 g, 2.59 mmol) and tri­ethyl­amine (0.34 mL, 0.25 g, 2.45 mmol) in CH_2_Cl_2_ (15 mL). After stirring at room temperature for 12 h, the solvent was evaporated under reduced pressure. The remaining white solid was washed several times with water, again suspended in CH_2_Cl_2_, filtered off and dried. Yield: 0.19 g (77%). ^1^H NMR (500 MHz, DMSO-*d*_6_, ppm): *δ* = 0.58–0.61 (*m*, 6H, CH_2_), 0.70–0.74 (*m*, 6H, CH_2_), 2.85–2.91 (*m*, 3H, CH), 8.31 (*s*, 3H, ar­yl), 8.65 (*d*, 3H, *J* = 4.1 Hz, NH). ^13^C NMR (125 MHz, DMSO-*d*_6_, ppm): *δ* = 5.7 (CH_2_), 23.2 (CH), 128.4 (ar­yl), 134.8 (ar­yl), 166.8 (C=O). MS (ESI): *m*/*z* calculated for C_18_H_22_N_3_O_3_: 328.2 [*M* + H]^+^, found 328.1. Single crystals suitable for X-ray diffraction were obtained by crystallization of the title compound from DMSO.

## 6. Refinement

Crystal data, data collection and structure refinement details are summarized in Table 2 ▸. The non-hydrogen atoms were refined anisotropically. All C-bound hydrogen atoms were positioned geometrically and refined isotropically using the riding model with C—H = 0.99–1.00 Å (cyclo­alk­yl), 0.95 Å (ar­yl); *U*_iso_(H) = 1.2–1.5*U*_eq_(C). The positions of the N—H hydrogens could be located in difference-Fourier maps and were refined to a target value of 0.90 Å [*U*_iso_(H) = 1.2*U*_eq_(N)].

## Supplementary Material

Crystal structure: contains datablock(s) I. DOI: 10.1107/S2056989024009800/ex2087sup1.cif

Structure factors: contains datablock(s) I. DOI: 10.1107/S2056989024009800/ex2087Isup2.hkl

Supporting information file. DOI: 10.1107/S2056989024009800/ex2087Isup3.cml

CCDC reference: 2389250

Additional supporting information:  crystallographic information; 3D view; checkCIF report

## Figures and Tables

**Figure 3:**
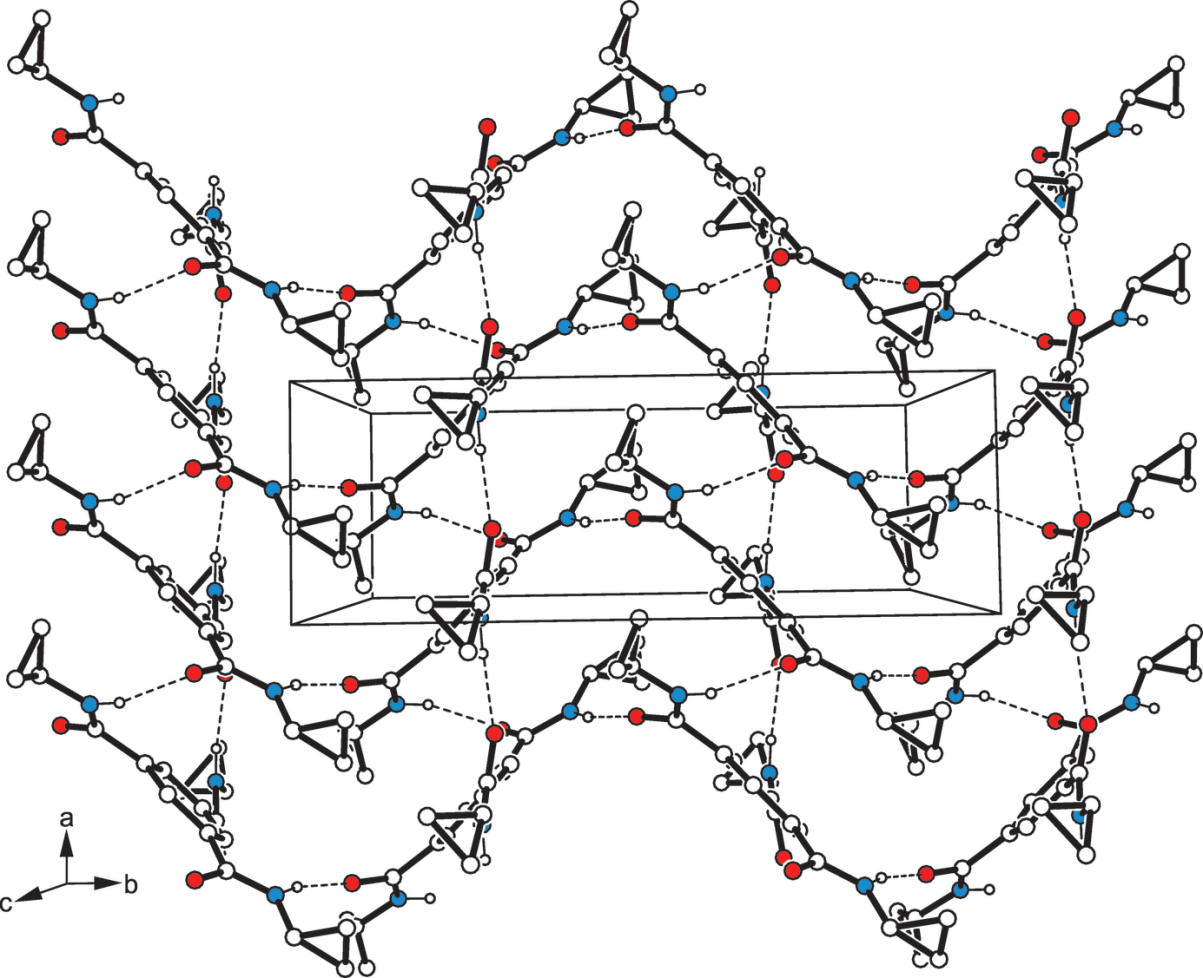
Packing diagram of the title compound showing the two-dimensional supra­molecular network. Dashed lines indicate N—H⋯O hydrogen bonds.

**Table 1:** Hydrogen-bond geometry (Å, °)

*D*—H⋯*A*	*D*—H	H⋯*A*	*D*⋯*A*	*D*—H⋯*A*
N1—H1*A*⋯O3^i^	0.90 (1)	1.96 (1)	2.8471 (15)	168 (1)
N2—H2*A*⋯O2^ii^	0.89 (1)	1.96 (1)	2.8253 (13)	165 (2)
N3—H3*A*⋯O1^ii^	0.89 (1)	2.04 (1)	2.8937 (14)	159 (1)
C2—H2⋯O3^i^	0.95	2.58	3.3485 (17)	139
C14—H14*A*⋯O1^iii^	0.99	2.55	3.2839 (17)	131

Symmetry codes: (i) 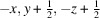; (ii) 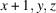; (iii) 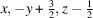.

**Table 2:** Experimental details

Crystal data	
Chemical formula	C_18_H_21_N_3_O_3_
*M*_r_	327.38
Crystal system, space group	Monoclinic, *P*2_1_/*c*
Temperature (K)	100
*a*, *b*, *c* (Å)	4.9435 (2), 14.2798 (5), 23.1247 (9)
β (°)	95.512 (2)
*V* (Å^3^)	1624.87 (11)
*Z*	4
Radiation type	Mo *K*α
μ (mm^−1^)	0.09
Crystal size (mm)	0.47 × 0.11 × 0.08

Data collection	
Diffractometer	Bruker CCD area detector
Absorption correction	Multi-scan (*SADABS*; Krause *et al.*, 2015 ▸)
*T*_min_, *T*_max_	0.958, 0.993
No. of measured, independent and observed [*I* > 2σ(*I*)] reflections	15717, 3779, 3059
*R*_int_	0.028
(sin θ/λ)_max_ (Å^−1^)	0.652

Refinement	
*R*[*F*^2^ > 2σ(*F*^2^)], *wR*(*F*^2^), *S*	0.040, 0.101, 1.02
No. of reflections	3779
No. of parameters	257
No. of restraints	63
H-atom treatment	H atoms treated by a mixture of independent and constrained refinement
Δρ_max_, Δρ_min_ (e Å^−3^)	0.36, −0.22

Computer programs: *APEX2* and *SAINT* (Bruker, 2014 ▸), *SHELXS97* and *SHELXTL* (Sheldrick, 2008 ▸), *SHELXL2014/7* (Sheldrick, 2015 ▸) and *ORTEP-3 for Windows* (Farrugia, 2012 ▸).

## supplementary crystallographic information

### N,N',N''-Tricyclopropylbenzene-1,3,5-tricarboxamide Crystal data

**Table:**

C_18_H_21_N_3_O_3_	*F*(000) = 696
*M**_r_* = 327.38	*D*_x_ = 1.338 Mg m^−^^3^
Monoclinic, *P*2_1_/*c*	Mo *K*α radiation, λ = 0.71073 Å
*a* = 4.9435 (2) Å	Cell parameters from 4588 reflections
*b* = 14.2798 (5) Å	θ = 2.9–28.5°
*c* = 23.1247 (9) Å	µ = 0.09 mm^−^^1^
β = 95.512 (2)°	*T* = 100 K
*V* = 1624.87 (11) Å^3^	Needle, colourless
*Z* = 4	0.47 × 0.11 × 0.08 mm

### N,N',N''-Tricyclopropylbenzene-1,3,5-tricarboxamide Data collection

**Table:**

Bruker CCD area detector diffractometer	3059 reflections with *I* > 2σ(*I*)
phi and ω scans	*R*_int_ = 0.028
Absorption correction: multi-scan (SADABS; Krause *et al.*, 2015)	θ_max_ = 27.6°, θ_min_ = 1.7°
*T*_min_ = 0.958, *T*_max_ = 0.993	*h* = −6→6
15717 measured reflections	*k* = −18→18
3779 independent reflections	*l* = −30→30

### N,N',N''-Tricyclopropylbenzene-1,3,5-tricarboxamide Refinement

**Table:**

Refinement on *F*^2^	Primary atom site location: difference Fourier map
Least-squares matrix: full	Hydrogen site location: mixed
*R*[*F*^2^ > 2σ(*F*^2^)] = 0.040	H atoms treated by a mixture of independent and constrained refinement
*wR*(*F*^2^) = 0.101	*w* = 1/[σ^2^(*F*_o_^2^) + (0.043*P*)^2^ + 0.8263*P*] where *P* = (*F*_o_^2^ + 2*F*_c_^2^)/3
*S* = 1.02	(Δ/σ)_max_ < 0.001
3779 reflections	Δρ_max_ = 0.36 e Å^−^^3^
257 parameters	Δρ_min_ = −0.22 e Å^−^^3^
63 restraints	

### N,N',N''-Tricyclopropylbenzene-1,3,5-tricarboxamide Special details

**Table:**

Geometry. All esds (except the esd in the dihedral angle between two l.s. planes) are estimated using the full covariance matrix. The cell esds are taken into account individually in the estimation of esds in distances, angles and torsion angles; correlations between esds in cell parameters are only used when they are defined by crystal symmetry. An approximate (isotropic) treatment of cell esds is used for estimating esds involving l.s. planes.

### N,N',N''-Tricyclopropylbenzene-1,3,5-tricarboxamide Fractional atomic coordinates and isotropic or equivalent isotropic displacement parameters (Å^2^)

**Table:**

	*x*	*y*	*z*	*U*_iso_*/*U*_eq_	Occ. (<1)
O1	−0.31098 (19)	0.74373 (6)	0.39186 (4)	0.0168 (2)	
O2	−0.36865 (18)	0.74689 (7)	0.12301 (4)	0.0208 (2)	
O3	0.4118 (2)	0.48708 (7)	0.26455 (4)	0.0226 (2)	
N1	−0.4171 (2)	0.86911 (8)	0.33459 (5)	0.0159 (2)	
H1A	−0.396 (3)	0.9006 (11)	0.3017 (5)	0.027 (4)*	
N2	0.0624 (2)	0.72544 (8)	0.10159 (5)	0.0156 (2)	
H2A	0.238 (2)	0.7278 (12)	0.1146 (7)	0.024 (4)*	
N3	0.5354 (2)	0.55827 (8)	0.35031 (5)	0.0198 (3)	
H3A	0.550 (3)	0.6129 (8)	0.3690 (7)	0.029 (5)*	
C1	−0.1216 (3)	0.74915 (9)	0.30131 (6)	0.0138 (3)	
C2	−0.1799 (3)	0.76665 (9)	0.24228 (6)	0.0144 (3)	
H2	−0.3183	0.8101	0.2294	0.017*	
C3	−0.0353 (3)	0.72041 (9)	0.20210 (5)	0.0138 (3)	
C4	0.1643 (3)	0.65575 (9)	0.22084 (6)	0.0145 (3)	
H4	0.2630	0.6246	0.1933	0.017*	
C5	0.2204 (3)	0.63643 (9)	0.27977 (6)	0.0140 (3)	
C6	0.0787 (3)	0.68403 (9)	0.31969 (6)	0.0142 (3)	
H6	0.1187	0.6721	0.3600	0.017*	
C7	−0.2892 (3)	0.78815 (9)	0.34647 (6)	0.0141 (3)	
C8	−0.6025 (3)	0.90605 (9)	0.37302 (6)	0.0172 (3)	
H8	−0.6957	0.8585	0.3959	0.021*	
C9	−0.5473 (3)	0.99839 (11)	0.40248 (7)	0.0257 (3)	
H9A	−0.6011	1.0060	0.4424	0.031*	
H9B	−0.3803	1.0324	0.3946	0.031*	
C10	−0.7684 (3)	0.98916 (11)	0.35391 (7)	0.0242 (3)	
H10A	−0.9587	0.9913	0.3638	0.029*	
H10B	−0.7380	1.0177	0.3161	0.029*	
C11	−0.1271 (3)	0.73253 (9)	0.13884 (6)	0.0145 (3)	
C12	−0.0096 (3)	0.73106 (10)	0.03993 (5)	0.0157 (3)	
H12	−0.1448	0.7804	0.0266	0.019*	
C13	0.2084 (3)	0.71258 (10)	0.00118 (6)	0.0198 (3)	
H13A	0.3907	0.6955	0.0196	0.024*	
H13B	0.2087	0.7505	−0.0346	0.024*	
C14	−0.0184 (3)	0.64273 (10)	0.00452 (6)	0.0194 (3)	
H14A	−0.1573	0.6381	−0.0292	0.023*	
H14B	0.0248	0.5830	0.0251	0.023*	
C15	0.4014 (3)	0.55474 (9)	0.29743 (6)	0.0157 (3)	
C16	0.7030 (9)	0.4825 (4)	0.37374 (17)	0.0243 (9)	0.700 (7)
H16	0.7408	0.4319	0.3457	0.029*	0.700 (7)
C17	0.6653 (7)	0.4516 (3)	0.43355 (16)	0.0279 (7)	0.700 (7)
H17A	0.5344	0.4867	0.4551	0.034*	0.700 (7)
H17B	0.6748	0.3836	0.4418	0.034*	0.700 (7)
C18	0.9190 (7)	0.4996 (3)	0.42163 (18)	0.0384 (8)	0.700 (7)
H18A	1.0868	0.4616	0.4225	0.046*	0.700 (7)
H18B	0.9464	0.5647	0.4357	0.046*	0.700 (7)
C16A	0.6466 (18)	0.4719 (12)	0.3775 (4)	0.0234 (15)	0.300 (7)
H16A	0.5688	0.4117	0.3613	0.028*	0.300 (7)
C17A	0.7441 (19)	0.4723 (7)	0.4401 (3)	0.0296 (14)	0.300 (7)
H17C	0.7222	0.5308	0.4621	0.036*	0.300 (7)
H17D	0.7239	0.4139	0.4623	0.036*	0.300 (7)
C18A	0.9421 (12)	0.4725 (5)	0.3963 (4)	0.0313 (13)	0.300 (7)
H18C	1.0456	0.4142	0.3911	0.038*	0.300 (7)
H18D	1.0438	0.5311	0.3910	0.038*	0.300 (7)

### N,N',N''-Tricyclopropylbenzene-1,3,5-tricarboxamide Atomic displacement parameters (Å^2^)

**Table:**

	*U*^11^	*U*^22^	*U*^33^	*U*^12^	*U*^13^	*U*^23^
O1	0.0226 (5)	0.0171 (5)	0.0109 (4)	0.0015 (4)	0.0031 (4)	0.0008 (4)
O2	0.0123 (5)	0.0349 (6)	0.0150 (5)	0.0026 (4)	0.0010 (4)	0.0009 (4)
O3	0.0308 (6)	0.0183 (5)	0.0184 (5)	0.0057 (4)	0.0001 (4)	−0.0049 (4)
N1	0.0197 (6)	0.0160 (5)	0.0129 (5)	0.0025 (4)	0.0056 (4)	0.0011 (4)
N2	0.0113 (5)	0.0254 (6)	0.0102 (5)	0.0005 (4)	0.0008 (4)	0.0002 (4)
N3	0.0256 (6)	0.0165 (6)	0.0161 (6)	0.0064 (5)	−0.0035 (5)	−0.0024 (5)
C1	0.0147 (6)	0.0131 (6)	0.0138 (6)	−0.0020 (5)	0.0027 (5)	−0.0017 (5)
C2	0.0137 (6)	0.0150 (6)	0.0144 (6)	0.0000 (5)	0.0006 (5)	0.0006 (5)
C3	0.0132 (6)	0.0168 (6)	0.0114 (6)	−0.0028 (5)	0.0011 (5)	−0.0005 (5)
C4	0.0132 (6)	0.0170 (6)	0.0133 (6)	−0.0008 (5)	0.0023 (5)	−0.0026 (5)
C5	0.0139 (6)	0.0138 (6)	0.0139 (6)	−0.0008 (5)	−0.0001 (5)	−0.0006 (5)
C6	0.0165 (6)	0.0152 (6)	0.0107 (6)	−0.0017 (5)	0.0001 (5)	−0.0002 (5)
C7	0.0151 (6)	0.0152 (6)	0.0119 (6)	−0.0013 (5)	0.0004 (5)	−0.0021 (5)
C8	0.0181 (6)	0.0176 (7)	0.0168 (7)	0.0019 (5)	0.0070 (5)	0.0003 (5)
C9	0.0220 (7)	0.0275 (8)	0.0287 (8)	0.0001 (6)	0.0075 (6)	−0.0113 (6)
C10	0.0227 (7)	0.0253 (7)	0.0255 (8)	0.0083 (6)	0.0068 (6)	0.0026 (6)
C11	0.0148 (6)	0.0166 (6)	0.0120 (6)	−0.0005 (5)	0.0013 (5)	−0.0002 (5)
C12	0.0157 (6)	0.0212 (7)	0.0103 (6)	0.0015 (5)	0.0013 (5)	0.0009 (5)
C13	0.0192 (7)	0.0273 (7)	0.0132 (6)	−0.0021 (5)	0.0039 (5)	−0.0022 (5)
C14	0.0227 (7)	0.0209 (7)	0.0143 (6)	−0.0018 (5)	−0.0004 (5)	−0.0005 (5)
C15	0.0176 (6)	0.0148 (6)	0.0150 (6)	0.0009 (5)	0.0031 (5)	−0.0003 (5)
C16	0.0310 (18)	0.0239 (19)	0.0174 (11)	0.0150 (16)	−0.0016 (12)	0.0002 (10)
C17	0.0242 (15)	0.0277 (15)	0.0317 (14)	0.0028 (11)	0.0015 (11)	0.0114 (11)
C18	0.0327 (14)	0.0422 (17)	0.0365 (17)	−0.0049 (12)	−0.0162 (13)	0.0200 (13)
C16A	0.022 (3)	0.019 (3)	0.028 (3)	0.003 (2)	−0.003 (2)	0.002 (2)
C17A	0.032 (3)	0.029 (3)	0.027 (2)	0.010 (2)	−0.002 (2)	0.007 (2)
C18A	0.022 (2)	0.027 (2)	0.044 (3)	0.0003 (19)	0.001 (2)	0.015 (2)

### N,N',N''-Tricyclopropylbenzene-1,3,5-tricarboxamide Geometric parameters (Å, º)

**Table:**

O1—C7	1.2400 (16)	C9—H9A	0.9900
O2—C11	1.2321 (16)	C9—H9B	0.9900
O3—C15	1.2337 (16)	C10—H10A	0.9900
N1—C7	1.3334 (17)	C10—H10B	0.9900
N1—C8	1.4369 (16)	C12—C13	1.4896 (18)
N1—H1A	0.897 (9)	C12—C14	1.5023 (19)
N2—C11	1.3361 (16)	C12—H12	1.0000
N2—C12	1.4381 (16)	C13—C14	1.5080 (19)
N2—H2A	0.893 (9)	C13—H13A	0.9900
N3—C15	1.3346 (17)	C13—H13B	0.9900
N3—C16	1.437 (6)	C14—H14A	0.9900
N3—C16A	1.467 (15)	C14—H14B	0.9900
N3—H3A	0.891 (9)	C16—C17	1.481 (5)
C1—C2	1.3903 (18)	C16—C18	1.483 (4)
C1—C6	1.3948 (18)	C16—H16	1.0000
C1—C7	1.5010 (17)	C17—C18	1.479 (4)
C2—C3	1.3921 (18)	C17—H17A	0.9900
C2—H2	0.9500	C17—H17B	0.9900
C3—C4	1.3899 (18)	C18—H18A	0.9900
C3—C11	1.4996 (18)	C18—H18B	0.9900
C4—C5	1.3921 (18)	C16A—C17A	1.481 (9)
C4—H4	0.9500	C16A—C18A	1.483 (8)
C5—C6	1.3889 (18)	C16A—H16A	1.0000
C5—C15	1.5026 (18)	C17A—C18A	1.474 (8)
C6—H6	0.9500	C17A—H17C	0.9900
C8—C10	1.4854 (19)	C17A—H17D	0.9900
C8—C9	1.497 (2)	C18A—H18C	0.9900
C8—H8	1.0000	C18A—H18D	0.9900
C9—C10	1.496 (2)		
			
C7—N1—C8	120.59 (11)	C13—C12—C14	60.53 (9)
C7—N1—H1A	121.2 (11)	N2—C12—H12	116.1
C8—N1—H1A	118.2 (11)	C13—C12—H12	116.1
C11—N2—C12	120.85 (11)	C14—C12—H12	116.1
C11—N2—H2A	120.0 (11)	C12—C13—C14	60.15 (9)
C12—N2—H2A	118.2 (11)	C12—C13—H13A	117.8
C15—N3—C16	122.3 (2)	C14—C13—H13A	117.8
C15—N3—C16A	119.6 (5)	C12—C13—H13B	117.8
C15—N3—H3A	119.2 (12)	C14—C13—H13B	117.8
C16—N3—H3A	117.3 (12)	H13A—C13—H13B	114.9
C16A—N3—H3A	121.2 (13)	C12—C14—C13	59.32 (9)
C2—C1—C6	119.55 (12)	C12—C14—H14A	117.8
C2—C1—C7	122.69 (12)	C13—C14—H14A	117.8
C6—C1—C7	117.28 (11)	C12—C14—H14B	117.8
C1—C2—C3	119.91 (12)	C13—C14—H14B	117.8
C1—C2—H2	120.0	H14A—C14—H14B	115.0
C3—C2—H2	120.0	O3—C15—N3	123.22 (13)
C4—C3—C2	120.12 (12)	O3—C15—C5	119.98 (12)
C4—C3—C11	121.33 (11)	N3—C15—C5	116.73 (11)
C2—C3—C11	118.02 (12)	N3—C16—C17	117.2 (4)
C3—C4—C5	120.37 (12)	N3—C16—C18	120.4 (4)
C3—C4—H4	119.8	C17—C16—C18	59.9 (2)
C5—C4—H4	119.8	N3—C16—H16	115.9
C6—C5—C4	119.21 (12)	C17—C16—H16	115.9
C6—C5—C15	121.63 (12)	C18—C16—H16	115.9
C4—C5—C15	118.51 (11)	C18—C17—C16	60.1 (2)
C5—C6—C1	120.82 (12)	C18—C17—H17A	117.8
C5—C6—H6	119.6	C16—C17—H17A	117.8
C1—C6—H6	119.6	C18—C17—H17B	117.8
O1—C7—N1	122.67 (12)	C16—C17—H17B	117.8
O1—C7—C1	119.86 (12)	H17A—C17—H17B	114.9
N1—C7—C1	117.43 (11)	C17—C18—C16	60.0 (2)
N1—C8—C10	118.44 (12)	C17—C18—H18A	117.8
N1—C8—C9	120.37 (12)	C16—C18—H18A	117.8
C10—C8—C9	60.20 (10)	C17—C18—H18B	117.8
N1—C8—H8	115.5	C16—C18—H18B	117.8
C10—C8—H8	115.5	H18A—C18—H18B	114.9
C9—C8—H8	115.5	N3—C16A—C17A	119.3 (12)
C10—C9—C8	59.51 (10)	N3—C16A—C18A	116.1 (9)
C10—C9—H9A	117.8	C17A—C16A—C18A	59.6 (4)
C8—C9—H9A	117.8	N3—C16A—H16A	116.6
C10—C9—H9B	117.8	C17A—C16A—H16A	116.6
C8—C9—H9B	117.8	C18A—C16A—H16A	116.6
H9A—C9—H9B	115.0	C18A—C17A—C16A	60.3 (4)
C8—C10—C9	60.29 (10)	C18A—C17A—H17C	117.7
C8—C10—H10A	117.7	C16A—C17A—H17C	117.7
C9—C10—H10A	117.7	C18A—C17A—H17D	117.7
C8—C10—H10B	117.7	C16A—C17A—H17D	117.7
C9—C10—H10B	117.7	H17C—C17A—H17D	114.9
H10A—C10—H10B	114.9	C17A—C18A—C16A	60.1 (4)
O2—C11—N2	122.73 (12)	C17A—C18A—H18C	117.8
O2—C11—C3	120.18 (11)	C16A—C18A—H18C	117.8
N2—C11—C3	117.07 (11)	C17A—C18A—H18D	117.8
N2—C12—C13	117.52 (11)	C16A—C18A—H18D	117.8
N2—C12—C14	119.02 (11)	H18C—C18A—H18D	114.9
			
C6—C1—C2—C3	−1.09 (19)	C4—C3—C11—O2	141.84 (13)
C7—C1—C2—C3	−173.04 (12)	C2—C3—C11—O2	−29.81 (18)
C1—C2—C3—C4	0.99 (19)	C4—C3—C11—N2	−37.08 (18)
C1—C2—C3—C11	172.74 (11)	C2—C3—C11—N2	151.28 (12)
C2—C3—C4—C5	0.26 (19)	C11—N2—C12—C13	−173.74 (12)
C11—C3—C4—C5	−171.21 (12)	C11—N2—C12—C14	−103.94 (15)
C3—C4—C5—C6	−1.40 (19)	N2—C12—C13—C14	109.50 (13)
C3—C4—C5—C15	169.51 (12)	N2—C12—C14—C13	−107.07 (13)
C4—C5—C6—C1	1.30 (19)	C16—N3—C15—O3	−0.5 (3)
C15—C5—C6—C1	−169.32 (12)	C16A—N3—C15—O3	−15.6 (5)
C2—C1—C6—C5	−0.06 (19)	C16—N3—C15—C5	176.5 (2)
C7—C1—C6—C5	172.32 (12)	C16A—N3—C15—C5	161.4 (5)
C8—N1—C7—O1	−4.0 (2)	C6—C5—C15—O3	142.42 (13)
C8—N1—C7—C1	173.74 (11)	C4—C5—C15—O3	−28.26 (18)
C2—C1—C7—O1	149.28 (13)	C6—C5—C15—N3	−34.62 (18)
C6—C1—C7—O1	−22.83 (18)	C4—C5—C15—N3	154.70 (12)
C2—C1—C7—N1	−28.51 (18)	C15—N3—C16—C17	−132.1 (4)
C6—C1—C7—N1	159.38 (12)	C15—N3—C16—C18	158.6 (3)
C7—N1—C8—C10	−171.59 (13)	N3—C16—C17—C18	−111.1 (4)
C7—N1—C8—C9	118.15 (15)	N3—C16—C18—C17	105.8 (4)
N1—C8—C9—C10	107.48 (14)	C15—N3—C16A—C17A	−167.5 (7)
N1—C8—C10—C9	−110.63 (14)	C15—N3—C16A—C18A	124.3 (8)
C12—N2—C11—O2	−2.3 (2)	N3—C16A—C17A—C18A	−104.8 (9)
C12—N2—C11—C3	176.63 (12)	N3—C16A—C18A—C17A	110.2 (12)

### N,N',N''-Tricyclopropylbenzene-1,3,5-tricarboxamide Hydrogen-bond geometry (Å, º)

**Table:**

*D*—H···*A*	*D*—H	H···*A*	*D*···*A*	*D*—H···*A*
N1—H1*A*···O3^i^	0.90 (1)	1.96 (1)	2.8471 (15)	168 (1)
N2—H2*A*···O2^ii^	0.89 (1)	1.96 (1)	2.8253 (13)	165 (2)
N3—H3*A*···O1^ii^	0.89 (1)	2.04 (1)	2.8937 (14)	159 (1)
C2—H2···O3^i^	0.95	2.58	3.3485 (17)	139
C14—H14*A*···O1^iii^	0.99	2.55	3.2839 (17)	131

Symmetry codes: (i) −*x*, *y*+1/2, −*z*+1/2; (ii) *x*+1, *y*, *z*; (iii) *x*, −*y*+3/2, *z*−1/2.
